# Implementation Method of Five-Axis CNC RTOS Kernel Based on gLink-II Bus

**DOI:** 10.3390/s25102960

**Published:** 2025-05-08

**Authors:** Liangji Chen, Hansong Gao, Huiying Li, Haohao Xu

**Affiliations:** Key Laboratory of Advanced Manufacturing and Automation Technology (Guilin University of Technology), Education Department of Guangxi Zhuang Autonomous Region, Guilin 541006, China; chenliangji@glut.edu.cn (L.C.); ghs0730@outlook.com (H.G.);

**Keywords:** gLink-II bus protocol, five-axis linkage, CNC system, direct linear interpolation, acceleration and deceleration planning, real-time performance

## Abstract

With the rapid development of Computerized Numerical Control (CNC) systems, traditional industrial communication protocols fail to meet the requirements for high real-time performance and reliability. To address these challenges, an open five-axis CNC system is designed and implemented based on the gLink-II bus protocol. This system features a layered architecture that integrates the Windows operating system with a Real-Time Operating System (RTOS) kernel, along with a multithreaded data interaction structure based on a circular buffer to enhance real-time data transmission performance and improve system responsiveness. In the direct linear interpolation control for five-axis machining, an acceleration and deceleration planning method is introduced, taking into account the kinematic constraints of the rotary axes. This method optimizes velocity and acceleration control. The experimental results show that the system achieves a maximum response error of less than 0.2 milliseconds and an interpolation period of less than 0.5 milliseconds in five-axis coordinated control. The system is capable of efficiently performing data processing and task scheduling, ensuring the stability of the CNC machining process.

## 1. Introduction

The rapid development of CNC technology has placed increased demands on the performance and reliability of motion control networks. With faster transmission speeds, extended transmission distances, improved anti-interference capabilities, and enhanced real-time control, a new generation of industrial communication technologies is gradually replacing traditional fieldbus systems. Protocols such as SERCOS, EtherCAT, PROFINET, and gLink-II have been successively introduced, significantly enhancing industrial communication performance [1,2,3]. However, some limitations still exist in the mainstream protocols. SERCOS suffers from poor compatibility and high maintenance costs, and EtherCAT cannot support direct communication between slave devices, and interruptions or poor connections severely affect its reliability [4,5,6]. To address these challenges, a five-axis linkage CNC system developed based on the gLink-II bus protocol effectively mitigates these drawbacks.

To address the issue of non-standard interfaces in legacy CNC systems, Martinez-Ruedas [7] designed a unified communication system based on the OPC UA protocol, which enhanced the monitoring and control capabilities of the equipment. However, its openness may be somewhat limited. Building on this, Zhou et al. [8] proposed an optimized data interaction architecture based on the SERCOS protocol, which improved data reading efficiency and reduced memory fragmentation through a layered structure and data filtering algorithms. Nevertheless, its adaptability to complex networked manufacturing environments still requires further enhancement. Liu et al. [9] employed an IPC + PMAC dual-CPU architecture, integrating technologies such as singularity avoidance, feedforward control, and error compensation to reduce machining errors. However, the system depends on specific hardware platforms. Ahmad et al. [10] proposed an open CNC monitoring system based on SOA-IoT, capable of monitoring cutting tool temperature, vibration, and current, thereby improving tool wear detection capabilities. However, its adaptability to multi-device access and big data processing is limited. Hatem et al. [11] introduced a CNC path optimization method based on the ACO algorithm, which optimizes G-code paths to reduce tool idle travel time in an open architecture control system. However, the method cannot optimize hybrid machining paths that involve both milling and drilling simultaneously. The aforementioned studies mainly focus on the functional development or data interaction optimization of open-architecture CNC systems but generally suffer from a strong dependence on specific hardware platforms or devices.

To address the scalability limitations of the aforementioned CNC systems, Liu et al. [12] applied the Dependency Inversion Principle and Component-Based Software Development architecture to achieve modularity and reconfigurability of CNC functions. However, the system suffers from high coupling among heterogeneous components. Wojtulewicz et al. [13] proposed a predictive maintenance control system based on the IIoT, which integrates data from CNC and robot controllers, enabling remote monitoring and enhancing fault prediction capability. However, the system demonstrates weak robustness under complex operating conditions. Zivanovic et al. [14] introduced a flexible programming and verification method for reconfigurable CNC systems, integrating CAD/CAM, STEP-NC standards, and the LinuxCNC control system to improve cross-platform compatibility and virtual simulation capability. Nonetheless, the system is primarily designed for woodworking applications. To meet dynamic adaptability requirements, Yuan-Ming, Dimic et al. [15,16] proposed a dynamically reconfigurable open-architecture CNC system, improving system flexibility and machining efficiency through modular design and virtual commissioning technology. However, this system exhibits slow real-time response. In conclusion, while previous studies have improved the openness of CNC systems, this often comes at the cost of reduced real-time performance.

In the field of industrial fieldbus, to enhance the real-time performance of the system, Zhang, Pei et al. [17,18] developed an EtherCAT master system based on the IgH open-source framework and an embedded platform, achieving low-latency and deterministic end-to-end communication. However, the system exhibits certain limitations in stability when handling high-concurrency data transmission scenarios. Adelt, Yoo et al. [19,20] proposed an abstract modeling and inter-task communication optimization method for key RTOS components, which reduces the overhead of synchronization operations, such as semaphores and mutexes. However, their approach lacks engineering validation in practical industrial control environments. Building upon this, Jeon, Zhou, Yan, Huang et al. [21,22,23,24] integrated artificial intelligence with real-time performance by developing machine tool network systems or edge intelligence architectures based on deep learning, neural networks, and digital twin technologies. These systems aim to achieve machine tool status perception, data processing, and the optimization of key performance indicators, though they still face challenges such as complex system architecture. In summary, while current studies have the advanced intelligence and industrial connectivity capabilities of CNC systems, they may still encounter issues related to complex system architecture and limited resource scheduling capabilities.

The interpolation algorithm plays a crucial role in CNC systems. To address the insufficient smoothness at the junction points between adjacent segments in interpolation algorithms, Li, Wan et al. [25,26] proposed a real-time look-ahead interpolation and corner smoothing algorithm, which enhances the continuity of motion trajectories. However, this method may have limited adaptive adjustment capabilities in scenarios involving short line segments. In response to the issues of low interpolation accuracy and inadequate simulation capabilities in CNC systems, Yang, Zhang et al. [27,28] implemented an affine transformation for coordinate conversion and integrated a virtual simulation platform for trajectory visualization. However, their work is primarily focused on simulation verification. Yang et al. [29] introduced a kinematics modeling and trajectory interpolation algorithm for CNC turning, which maintains a constant cutting angle and uniform feed rate, thereby reducing fluctuations in cutting force. However, this approach is specifically designed for certain non-circular machining equipment. While the above studies optimized motion trajectories, interpolation calculations were performed after post-processing, which could result in the loss of openness in the tool path file. Varga et al. [30] conducted a systematic study on the effects of typical finishing strategies, including Constant Z, spiral, and spiral circular tool paths, on surface quality in complex surface machining. Through experimental comparisons, the study analyzed the variations in surface topography, surface roughness, and machining errors under different tool path strategies, providing valuable engineering insights for process optimization and quality control in complex surface manufacturing.

To address the issues of slow response and poor smoothness in traditional acceleration and deceleration control, Li et al. [31] proposed a path planning method based on two-stage adaptive acceleration and deceleration control. They further improved motion smoothness and positioning accuracy through discretizing the acceleration and deceleration processes and optimizing the interpolation period [32], although their focus was mainly on single-axis path planning. Han et al. [33] employed cubic B-splines to smooth the corners of short linear segments, combining look-ahead interpolation and a seven-segment S-curve acceleration and deceleration control to achieve C² continuous tool trajectories, thereby reducing trajectory oscillations and machining errors. However, this method still relies on fitting G01 discrete linear segments. Tsai et al. [34] introduced an integrated dynamic acceleration and deceleration control method that combines pre-interpolation (ADBI) and post-interpolation (ADAI) acceleration and deceleration to reduce contour errors. However, it mainly relies on speed adjustments of the interpolation segments. The studies above may not have fully considered the constraints imposed by rotary axes on the translational axes of the machine tool during five-axis machining.

In conclusion, this paper presents a five-axis CNC system platform based on the gLink-II bus protocol. A direct linear interpolation method is employed for five-axis tool path machining, which is integrated into the CNC program. This allows interpolation planning to be performed before post-processing, thereby maintaining the openness of the tool path file. Considering the constraints of the rotary axes, acceleration and deceleration are planned during the direct linear interpolation process based on the speed and acceleration limits of the rotary axes. Additionally, the producer–consumer model is applied for data exchange via a ring buffer, enhancing the real-time data transmission performance in the RTOS.

## 2. Materials

### 2.1. The Topology Structure of the gLink-II Bus Numerical Control System

**Figure 1:**
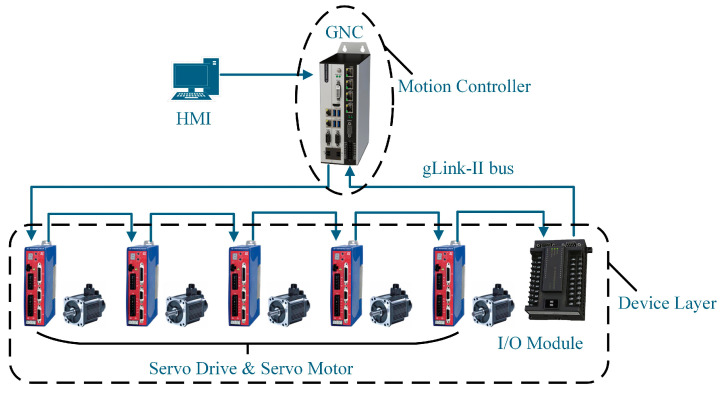
Topology structure of CNC system.

The topology of a CNC system plays a crucial role in determining its real-time performance, scalability, and capability for intelligent functionality [35,36,37,38,39,40]. Figure 1 illustrates the topology of the CNC system. It utilizes an embedded controller (GNC) based on the gLink-II bus to enable high-speed communication and precise motor control.

The system is developed using Visual Studio 2022, with the Human–Machine Interface (HMI) implemented in a Windows environment and real-time modules within the RTOS kernel. The HMI acts as the interactive interface for data exchange and parameter configuration. The GNC motion controller, which forms the core of the CNC system, handles tasks such as interpolation calculations and velocity preprocessing. High-speed data communication between the GNC and servo drives is facilitated by the gLink-II bus.

### 2.2. Configuration of System Closed-Loop Control Mode

To establish a motion control system using the gLink-II communication protocol on the Windows operating system, the configuration of the GNC motion controller is the first step. Initially, the controller driver (e.g., PCIe interface driver) must be installed, along with dynamic link libraries that contain controller instruction functions (e.g., core controller instruction .dll files and network initialization .dll files). Additionally, network configuration files (such as .rndma and .rnmap files) should be placed in the project directory of the development software.

Subsequently, hardware and software resources are integrated via the API provided by the controller within the Visual Studio (C++) integrated development environment. The gLink-II communication protocol facilitates the efficient data exchange and transmission of control instructions between hardware and software, thereby completing the system configuration.

**Figure 2:**
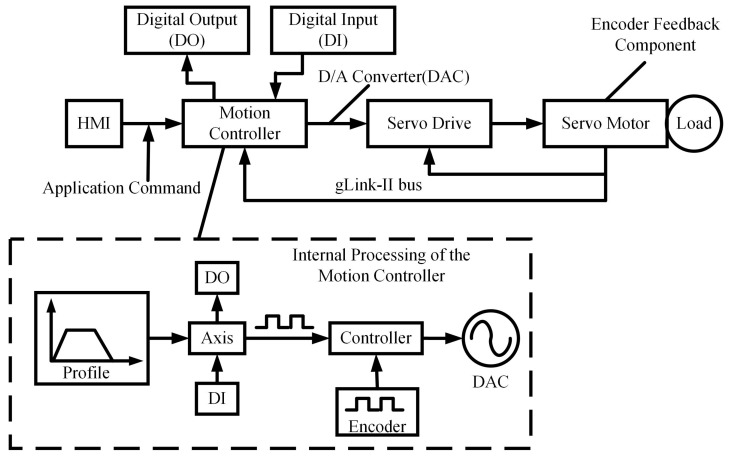
System composition and configuration.

As illustrated in Figure 2, the single-axis closed-loop control system integrates hardware components, such as the host computer, motion controller, and servo driver, via the gLink-II communication protocol. The host computer transmits motion planning instructions and I/O control commands, which are then converted by the motion controller into low-level motion commands and output to the servo driver in analog form.

The motion control module in the controller first carries out motion planning for the axis through interpolation calculations, generating trajectory profile positions. These positions then undergo post-processing before being entered into the Axis module. After equivalent output processing in the Axis module, the data are sent to the servo driver. Simultaneously, the controller continuously compares the planned position with the encoder counter in real time and generates a control output to the Digital-to-Analog Converter (DAC), enabling real-time closed-loop motor control.

The HMI of the host computer collects real-time axis data, including planned position, velocity, and acceleration, while simultaneously displaying feedback from the servo driver. This ensures the accuracy and real-time responsiveness of the closed-loop control.

## 3. Methods

**Figure 3:**
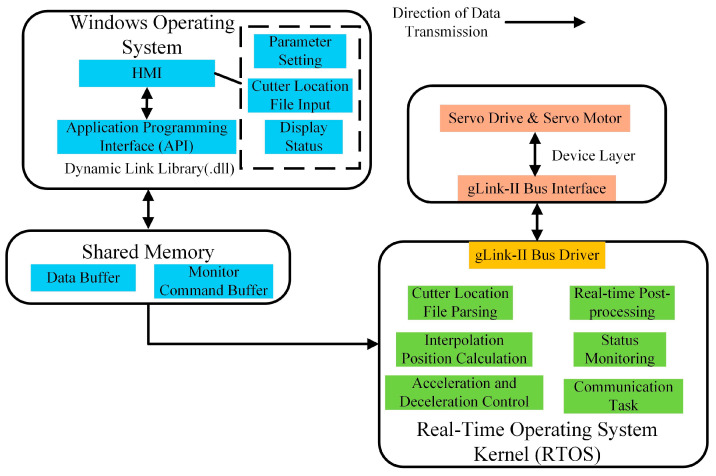
Layered system architecture.

A layered architecture is proposed for the CNC system, which includes the Windows operating system for human–machine interaction and an RTOS kernel for real-time control tasks. Data exchange between these two systems is achieved through Shared Memory, as shown in Figure 3.

The Windows operating system primarily manages the HMI functions, including parameter setting, cutter location file input, and display status. Tasks that require high precision and rapid response, such as interpolation position calculation, acceleration and deceleration control, and real-time post-processing, are handled by the RTOS to ensure real-time performance and reliability.

The Shared Memory module is divided into two buffers, the Data Buffer and the Monitor Command Buffer, each serving a distinct purpose. The Data Buffer facilitates bidirectional data exchange between the HMI and RTOS, while the Monitor Command Buffer tracks motor states, actual planned positions, and actual positions.

### 3.1. Implementation of Functions in RTOS Kernel

#### 3.1.1. Cutter Location File Parsing in RTOS

For a CNC system, the program header and footer must first be created according to the program format. Then, the cutter location file is read line by line. Each cutter code keyword is analyzed to determine whether coordinate transformation and code conversion are necessary [41,42,43,44].

Next, based on the correspondence between the cutter location code and the CNC program, each line of the cutter location code is converted into a CNC program. During the direct linear interpolation process for six-axis machining, six coordinate parameters (*x*, *y*, *z*, *i*, *j*, and *k*) from the cutter location code must be parsed. The parsed results are stored in a custom structure, std::vector<Position>, and ultimately, these instances are saved in the ‘coordinate’ vector and returned.

#### 3.1.2. Calculation of Interpolated Command Position Data

The current method for five-axis linear interpolation is primarily based on the structural design of five-axis CNC machines. It utilizes the post-processing module within CAM software to convert six parameters from the cutter location file—namely, cutter position coordinates and tool axis unit vectors (*x*, *y*, *z*, *i*, *j*, and *k*)—into five coordinate parameters corresponding to the three translational axes and two rotational axes [45,46,47,48].

While this approach remains the dominant method in modern CAM/CNC manufacturing systems, the .CLS format of the cutter location file is independent of the specific structure of five-axis machine tools, thus maintaining a degree of neutrality in terms of machine configuration. However, adopting this method may undermine the neutrality of the .CLS file format with respect to the machine structure.

This study takes an A-C dual-rotary head CNC machine as an example and directly performs linear interpolation calculations on the six parameters in the .CLS format [49]. The trapezoidal acceleration and deceleration of the translational axes are adjusted in accordance with the maximum permissible rotational velocity and angular acceleration of the A- and C-axes. After the real-time calculation of the target interpolated cutter location for each interpolation cycle, post-processing calculations are conducted to generate motion control command data for all five axes. The interpolated command data are then transmitted in real time to the hardware layer via bus communication.

We let the starting cutter position *P_s_*(*x_s_*, *y_s_*, *z_s_*, *i_s_*, *j_s_*, *k_s_*), which corresponds to the endpoint of the previously interpolated linear cutter trajectory, and the endpoint *P_e_*(*x_e_*, *y_e_*, *z_e_*, *i_e_*, *j_e_*, *k_e_*) of the linear cutter trajectory to be interpolated be obtained from the cutter location file. *T* is denoted as the interpolation cycle of the CNC system. The total number of interpolation cycles *n* required to interpolate the linear tool path is then given by the following equation:

(1) n=ceil(D/L)

In the equation, the function *ceil*() rounds up to the nearest integer. The term *D* represents the length of the linear cutter trajectory to be interpolated, while *L* is the interpolation step length.

According to the data sampling interpolation principle [50], a total of *n* + 1 interpolated machining cutter locations can be obtained from the starting cutter location to the endpoint through linear interpolation. The *m*-th interpolated machining cutter location *P_m_*(*x_m_*, *y_m_*, *z_m_*, *i_m_*, *j_m_*, *k_m_*) is given by

(2) $$\left\{\begin{array}{l} x_{m}=x_{s}+(x_{e}-x_{s})\times \frac{m}{n}\\ y_{m}=y_{s}+(y_{e}-y_{s})\times \frac{m}{n}\\ z_{m}=z_{s}+(z_{e}-z_{s})\times \frac{m}{n}\\ i_{m}=i_{s}+(i_{e}-i_{s})\times \frac{m}{n}\\ j_{m}=j_{s}+(j_{e}-j_{s})\times \frac{m}{n}\\ k_{m}=k_{s}+(k_{e}-k_{s})\times \frac{m}{n} \end{array}\right.\;m=0,1,2\ldots n$$

The interpolation position calculation module within the RTOS kernel uses the current cutter location *P_m_*(*x_m_*, *y_m_*, *z_m_*, *i_m_*, *j_m_*, *k_m_*) from Equation (2) as the interpolated command position data. There data are then subjected to acceleration and deceleration control, along with real-time post-processing. The resulting motion coordinate data for each axis are transmitted to the hardware layer to achieve position and motion control.

#### 3.1.3. Acceleration and Deceleration Control Based on Rotational Axis Kinematic Constraints

The trapezoidal acceleration and deceleration control method is favored for its compact code structure and ease of maintenance, making it a practical choice for velocity planning. In this CNC system, trapezoidal acceleration and deceleration are primarily employed to regulate the velocity. Furthermore, the velocity of the translational axes is adjusted based on the kinematic constraints of the rotational axes, ensuring synchronized motion between the translational and rotational axes, thus achieving effective five-axis linkage.

The system parameters include the initial and final composite velocities of the translational axes (*V_s_* and *V_e_*), the maximum allowable composite velocity and acceleration for the translational axes (*V_max_* and *a_max_*), and the maximum angular velocity and angular acceleration of the A-axis (*V_Amax_* and *a_Amax_*) and the C-axis (*V_Cmax_* and *a_Cmax_*). All maximum velocities and accelerations are positive values. Additionally, the starting and ending coordinates for the A-axis (*A_s_*, *A_e_*) and C-axis (*C_s_*, *C_e_*) are provided.

(1) Based on the Rotational Axis Maximum Angular Velocity Constraint

Assuming that, at a certain time period, *V_m_* represents the maximum composite velocity of the translational axes (*V_m_* ≤ *V_max_*), it can be derived [51,52] from the five-axis linear interpolation principle:

(3) $$\left\{\begin{array}{l} \Delta D_{m}=V_{m}\times T\\ \Delta A_{m}=V_{Am}\times T\\ \Delta C_{m}=V_{Cm}\times T \end{array}\right.$$

In this equation, Δ*D_m_*, Δ*A_m_*, and Δ*C_m_* denote the composite displacement of the translational axes, the angular displacement of the A-axis, and the angular displacement of the C-axis within the *m*-th interpolation cycle, respectively. *V_Am_* and *V_Cm_* represent the angular velocities of the A-axis and C-axis during the *m*-th cycle.

The linear distribution of Δ*D_m_*, Δ*A_m_*, and Δ*C_m_* based on Equation (3) is expressed as

(4) $$\frac{\Delta D_{m}}{D}=\frac{\Delta A_{m}}{A_{e}-A_{s}}=\frac{\Delta C_{m}}{C_{e}-C_{s}}$$

From Equations (3) and (4), it follows that when the translational axes reach the maximum velocity *V_m_*, the A-axis and C-axis achieve their maximum angular velocities, *V_Am_* and *V_Cm_*, which must not exceed their allowable limits (*V_Am_* ≤ *V_Amax_*, *V_Cm_* ≤ *V_Cmax_*). Furthermore, Equations (3) and (4) establish the relationship between the maximum velocity *V_m_* of the translational axes and the maximum angular velocities, *V_Am_* and *V_Cm_*, of the A-axis and C-axis, respectively:

(5) $$\left\{\begin{array}{l} V_{Am}=\frac{\Delta A_{m}}{T}=\frac{(A_{e}-A_{s})V_{m}}{D}\\ V_{Cm}=\frac{\Delta C_{m}}{T}=\frac{(C_{e}-C_{s})V_{m}}{D} \end{array}\right.$$

*V_Am_* is in the same direction as (*A_e_* − *A_s_*), and *V_Cm_* is in the same direction as (*C_e_* − *C_s_*). Therefore, Equation (5) can be written as

(6) $$\left\{\begin{array}{l} V_{Am}=sgn(V_{Am})abs(V_{Am})\\ V_{Cm}=sgn(V_{Cm})abs(V_{Cm}) \end{array}\right.$$

In this equation, *sgn*() represents the sign function, and *abs*() denotes the absolute value function.

The adjustment of the maximum composite velocity of the translational axes is classified into the following cases:

1) When *abs*(*V_Am_*) ≤ *V_Amax_* and *abs*(*V_Cm_*) ≤ *V_Cmax_*, this indicates that the angular velocities of the A and C rotational axes have not exceeded the allowable maximum angular velocity limits of the machine. In this case, no adjustment to the maximum composite velocity of the translational axes is required.

2) When *abs*(*V_Am_*) ≥ *V_Amax_* or *abs*(*V_Cm_*) ≥ *V_Cmax_*, this indicates that at least one of the A-axis or C-axis has exceeded the machine allowable maximum angular velocity limit. In this case, *sgn*(*V_Am_*)*V_Amax_* and *sgn*(*V_Cm_*)*V_Cmax_* are used to replace *V_Am_* and *V_Cm_* in Equation (5), resulting in Equation (7):

(7) $$\left\{\begin{array}{l} {\left(V_{m}\right)}_{A}=\frac{D\cdot sgn(V_{Am})V_{Amax}}{A_{e}-A_{s}}\\ {\left(V_{m}\right)}_{C}=\frac{D\cdot sgn(V_{Cm})V_{Cmax}}{C_{e}-C_{s}} \end{array}\right.$$

In Equation (7), (*V_m_*)*_A_* and (*V_m_*)*_C_* represent the maximum composite velocity of the translational axes after correction based on the allowable maximum angular velocities of the A- and C-axes. The smaller of the two values is denoted as (*V_m_*)′, which is obtained from Equation (8):

(8) $$\left(V_{m}\right)\prime =min\{{\left(V_{m}\right)}_{A},{\left(V_{m}\right)}_{C}\}$$

In this equation, *min*{} represents the function that selects the smaller value within the brackets.

Finally, the maximum composite velocity *V_max_* of the translational axes during trapezoidal acceleration and deceleration is reassigned. The value of (*V_m_*)′ from Equation (8) is assigned to *V_max_* for velocity planning.

(2) Based on Rotational Axis Maximum Angular Acceleration Constraint

In any *i*-th interpolation cycle, the relationship between the composite acceleration of the translational axes *a_i_* and the angular accelerations of the A-axis and C-axis, *a_Ai_* and *a_Ci_*, is given by Equation (9)

(9) $$\left\{\begin{array}{l} a_{Ai}=\frac{V_{A(i+1)}-V_{Ai}}{T}=\frac{a_{i}(A_{e}-A_{s})}{D}\\ a_{Ci}=\frac{V_{C(i+1)}-V_{Ci}}{T}=\frac{a_{i}(C_{e}-C_{s})}{D} \end{array}\right.$$

From Equation (9), it is evident that the composite acceleration of the translational axes *a_i_* is linear related to the angular accelerations of the A-axis and C-axis, *a_Ai_* and *a_Ci_* [53,54]. Thus, when the translational axes reach their maximum composite acceleration, *a_max_*, the A and C rotational axes also reach their respective maximum angular accelerations, (*a_A_*)*_max_* and (*a_C_*)*_max_*, as expressed in Equation (10) as follows:

(10) $$\left\{\begin{array}{l} {\left(a_{A}\right)}_{max}=abs(\frac{a_{max}(A_{e}-A_{s})}{D})\\ {\left(a_{C}\right)}_{max}=abs(\frac{a_{max}(C_{e}-C_{s})}{D}) \end{array}\right.$$

In this equation, (*a_A_*)*_max_* and (*a_C_*)*_max_* represent the actual maximum angular accelerations of the A-axis and C-axis, respectively.

The adjustment of the maximum composite acceleration of the translational axes is categorized into the following cases:

1) When (*a_A_*)*_max_* ≤ *a_Amax_* and (*a_C_*)*_max_* ≤ *a_Cmax_*, this indicates that the angular accelerations of both the A-axis and C-axis are within the machine allowable maximum angular acceleration limits. In this case, no adjustment to the maximum composite acceleration of the translational axes is necessary.

2) When (*a_A_*)*_max_* ≥ *a_Amax_* or (*a_C_*)*_max_* ≥ *a_Cmax_*, this indicates that at least one of the A-axis or C-axis has exceeded the machine allowable maximum angular acceleration limit. In this case, the values of *a_Amax_* and *a_Cmax_* replace (*a_A_*)*_max_* and (*a_C_*)*_max_* in Equation (10), resulting in Equation (11):

(11) $$\left\{\begin{array}{l} {\left(a_{max}\right)}_{A}=abs(\frac{D\cdot a_{Amax}}{A_{e}-A_{s}})\\ {\left(a_{max}\right)}_{C}=abs(\frac{D\cdot a_{Cmax}}{C_{e}-C_{s}}) \end{array}\right.$$

In Equation (11), (*a_max_*)*_A_* and (*a_max_*)*_C_* represent the maximum composite accelerations of the translational axes after being corrected based on the allowable maximum angular accelerations of the A- and C-axes. The smaller of these two values is denoted as (*a_max_*)′, which is calculated from Equation (12):

(12) $$\left(a_{max}\right)\prime =min\{{\left(a_{max}\right)}_{A},{\left(a_{max}\right)}_{C}\}$$

Subsequently, the maximum composite acceleration *a_max_* of the translational axes for trapezoidal acceleration and deceleration is reassigned. The value of (*a_max_*)′ from Equation (12) is used as the new value of *a_max_* for velocity planning.

#### 3.1.4. Real-Time Post-Processing of Interpolated Machining Cutter Locations

Post-processing is a crucial step in converting cutter location data files into machine-recognizable CNC system codes. Unlike traditional five-axis CNC machines, which first post-process the cutter location file into G-code before performing interpolation, the CNC system described here directly performs real-time post-processing after interpolation calculations and velocity planning on the six-coordinate parameters in the cutter location file.

For the AC dual-swing head five-axis CNC machine structure [50], after performing forward and inverse kinematics modeling, the six parameters (*x_m_*, *y_m_*, *z_m_*, *i_m_*, *j_m_*, and *k_m_*) are transformed into the machine coordinate system. These correspond to the three translational motion control coordinates, *X_m_*, *Y_m_*, and *Z_m_*, and the rotational motion control coordinates around the X-axis and Z-axis, *θ_Am_* and *θ_Cm_*. The coordinate transformation equations are provided in Equations (13) and (14).

(13) $$\left\{\begin{array}{l} \theta_{Am}=\arccos k_{m}\\ \theta_{Cm}=\left\{\begin{array}{l} \frac{\pi }{2}-\arctan\frac{i_{m}}{j_{m}}\;(i_{m}>0,j_{m}<0)\\ \frac{\pi }{2}+\arctan\frac{i_{m}}{j_{m}}\;(i_{m}>0,j_{m}>0)\\ \frac{3\pi }{2}-\arctan\frac{i_{m}}{j_{m}}\;(i_{m}<0,j_{m}<0)\\ \frac{3\pi }{2}+\arctan\frac{i_{m}}{j_{m}}\;(i_{m}<0,j_{m}>0) \end{array}\right. \end{array}\right.$$

(14) $$\left\{\begin{array}{l} X_{m}=x_{m}-L_{D}\sin\theta_{Am}\sin\theta_{Cm}\\ Y_{m}=y_{m}+L_{D}\sin\theta_{Am}\cos\theta_{Cm}\\ Z_{m}=z_{m}+L_{D}-L_{D}\cos\theta_{Am} \end{array}\right.$$

In these equations, *L_D_* represents the tool length relative to the rotational pivot point.

Additionally, trapezoidal acceleration and deceleration velocity planning is applied to the maximum composite velocity and acceleration of the translational axes. These values are adjusted according to the allowable maximum angular velocity and angular acceleration constraints of the A- and C-axes. The composite velocity and acceleration of the translational axes are then distributed across each axis based on the unit tool axis vector. This relationship is expressed in Equation (15).

(15) $$\left\{\begin{array}{l} {\left(V_{im}\right)}^{T}=V_{m}\cdot u^{T}\\ {\left(a_{im}\right)}^{T}=a_{m}\cdot u^{T} \end{array}\right.$$

In this equation, *V_im_* = (*V_xm_*, *V_ym_*, *V_zm_*) represents the velocities of the X, Y, and Z axes, respectively, and *a_im_* = (*a_xm_*, *a_ym_*, *a_zm_*) represents the accelerations of the X, Y, and Z axes, respectively. Here, *u* = (*i*, *j*, *k*) is the unit tool axis vector.

Taking the X-axis as an example, Equation (16) shows that once the position, velocity, and time of two adjacent data points are determined, the detailed motion pattern of each axis can be derived.

(16) $$\left\{\begin{array}{l} X_{m}=at^{3}+bt^{2}+ct+d\\ V_{xm}=3at^{2}+2bt+c\\ X_{m+1}=at^{3}+bt^{2}+ct+d\\ V_{xm+1}=3at^{2}+2bt+c \end{array}\right.$$

In a six-coordinate five-axis linkage control system, the primary control objective is to convert the interpolated spatial trajectory into corresponding position signals and velocity references for each motor axis, based on the trajectory planned by the host computer. After completing six-coordinate interpolation and post-processing, a series of discrete spatial data points are obtained, containing the absolute positions and corresponding velocities required by each motor axis at specified time points *T*. The processed spatiotemporal data points (position, velocity, and timing information) must be transmitted to the underlying motion controller via API functions, enabling the precise control of the motor velocity and position according to the time-sequenced commands.

### 3.2. Communication Tasks and Mechanisms in RTOS

To improve real-time performance in an RTOS, the producer–consumer multi-threading design pattern is implemented to address data synchronization and sharing across multiple threads.

The producer generates data or tasks and inserts them into a shared data structure, specifically a circular buffer. Within the system, this includes tasks such as reading and processing input data, parsing cutter location files, calculating six-coordinate interpolation positions, and managing acceleration and deceleration controls. After processing, the data are pushed in batches into the corresponding circular buffers for each axis. Once all tasks are completed, the buffer is marked as finished, signaling to the consumer thread that no further data will be produced.

The consumer thread retrieves data or tasks from the shared structure for further processing. Specifically, for each motor, the consumer fetches batches from its dedicated circular buffer and transmits them to the motion controller through API functions to control the motor. The consumer thread is assigned high priority to ensure that data retrieval and processing occur promptly, minimizing transmission latency and enhancing real-time performance.

**Figure 4:**
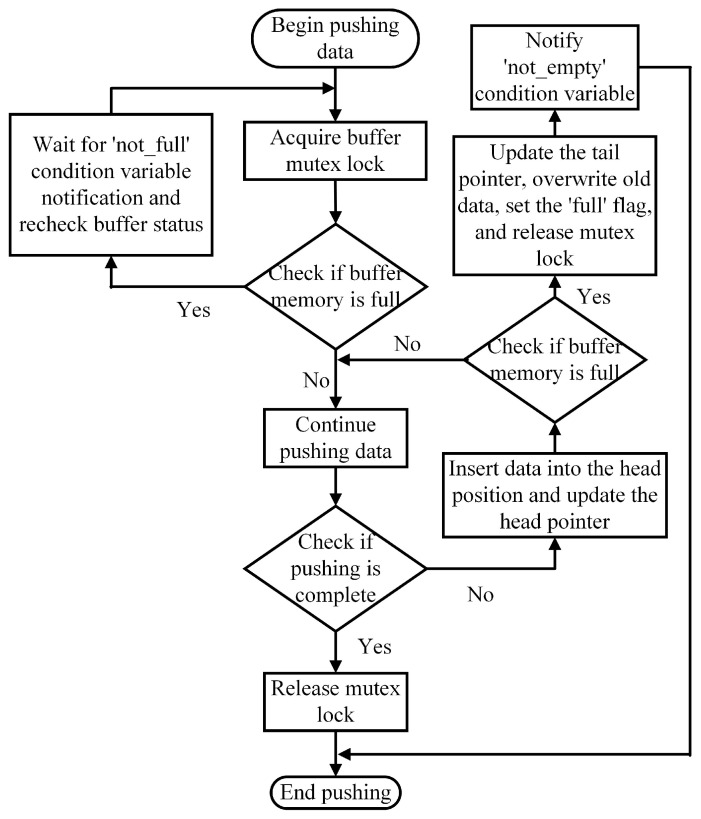
Producer circular buffer access mechanism.

The access mechanism for the producer’s circular buffer is shown in Figure 4. Initially, the producer acquires the mutex lock for the buffer and checks if the buffer is full.

If the buffer is full, the producer waits for a notification from the ‘not_full’ condition variable until space becomes available.

If there is space, the producer inserts data into the head of the buffer and updates the ‘head’ pointer.

The producer then checks the buffer again.

If the buffer is full, the producer updates the ‘tail’ pointer to overwrite the oldest data, sets the ‘full’ flag, releases the mutex lock, and signals to the consumer via the ‘not_empty’ condition variable that new data are available.

**Figure 5:**
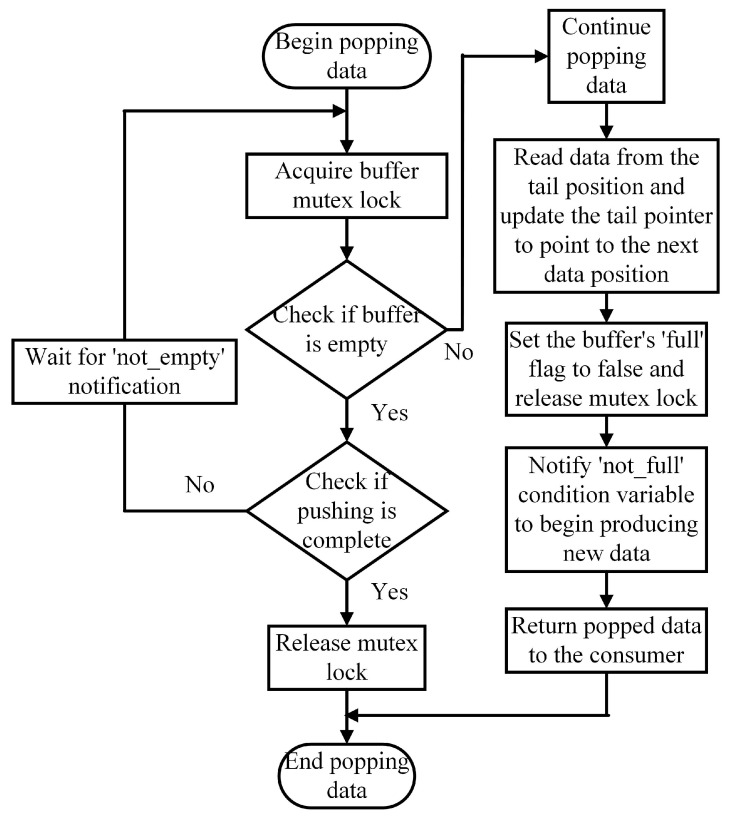
Consumer circular buffer access mechanism.

The access mechanism of the consumer circular buffer is depicted in Figure 5.

The consumer first acquires the mutex lock and checks if the buffer is empty.

If the buffer is empty, the consumer decides whether to release the lock and terminate based on the data push status or to wait for a notification from the ‘not_empty’ condition variable.

When the buffer contains data, the consumer retrieves it from the ‘tail’ position, updates the pointer, and clears the ‘full’ flag.

Finally, the consumer releases the lock and sends a signal to ‘not_full’, ensuring the safe and efficient retrieval and transmission of shared data by the consumer thread.

### 3.3. CNC System Program Execution and Data Flow Direction

**Figure 6:**
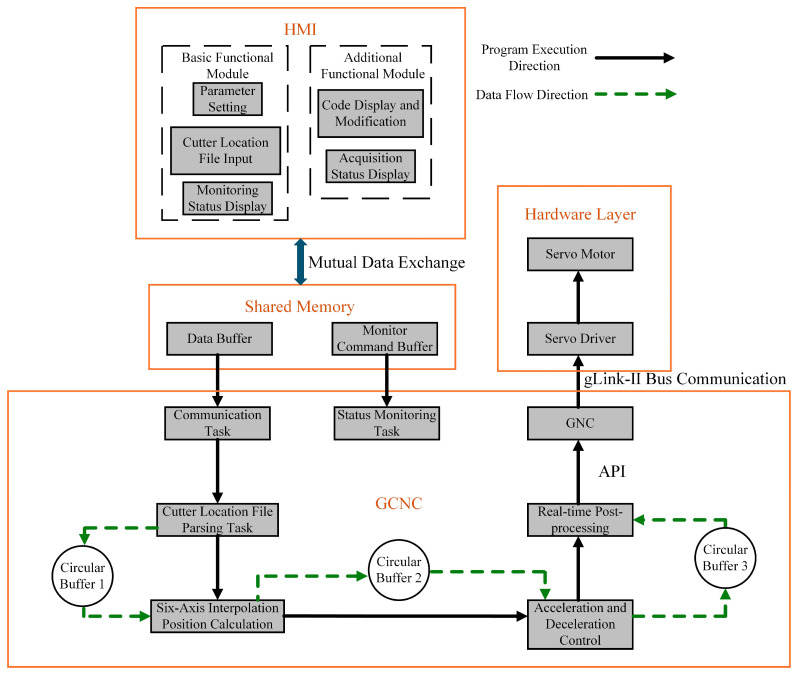
System program execution and data flow direction.

Figure 6 illustrates the program execution and data flow directions within the CNC system, organized in a hierarchical structure. The HMI, located at the top layer, operates within the Windows OS, while the GCNC, the core of the system, resides at the bottom layer. Data exchange between the HMI and GCNC is facilitated via Shared Memory.

The GCNC handles multiple tasks, including communication, the parsing of cutter location files, six-axis interpolation position calculations, acceleration/deceleration control, and real-time post-processing. The communication task initiates the system, with six-coordinate data extracted from the cutter location file stored in Circular Buffer 1. The system then processes these data by performing interpolation calculations, controlling acceleration and deceleration, and executing real-time post-processing. Data transmission between components is also managed using circular buffers.

When API functions such as GTN_PvtTable are invoked, communication with the hardware is established through drivers, writing PVT data points into the internal RAM of the controller. For each trajectory, a distinct PVT data table is allocated, where time, position, and velocity information is stored using specific data structures.

Upon calling the GTN_PvtStart API function, the controller firmware begins interpolation based on the time-sequenced data points stored in the table. It outputs control signals, formatted as pulse direction signals, to the motor driver. The firmware utilizes FPGA-based high-speed logic circuits within the motion controller, executing position and velocity closed-loop control algorithms at fixed intervals.

## 4. Results

### 4.1. Experimental Platform

**Figure 7:**
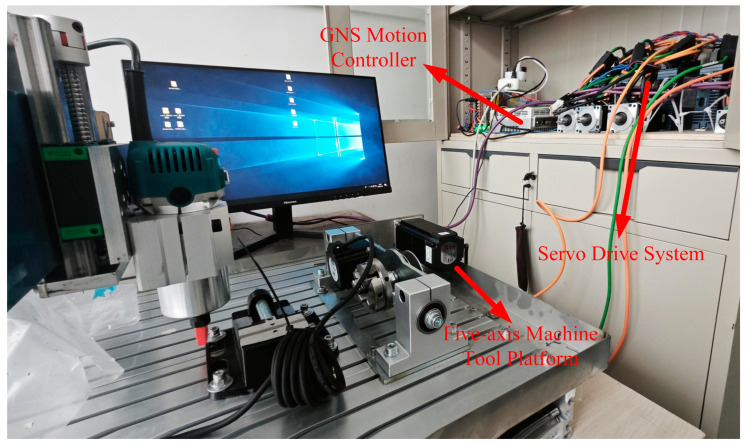
Servo motion platform.

The five-axis servo motion control platform developed in this study, as shown in Figure 7, is built on the gLink-II bus protocol. To validate the communication status and conduct motor speed simulation experiments, a five-axis machine tool model featuring an A–C dual rotary head is established.

**Figure 8:**
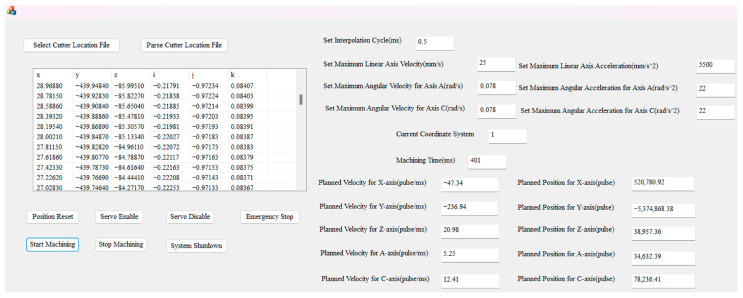
HMI.

An MFC-based CNC software system is developed to leverage the capabilities of the aforementioned CNC software, incorporating direct linear interpolation for six-coordinate cutter positions. The HMI, shown in Figure 8, includes essential functional modules, such as parameter settings and cutter location file parsing, in addition to RTOS tasks, including servo enable, emergency stop, and start machining.

### 4.2. Motor Speed Simulation Example

Based on the machine tool characteristics depicted in Figure 7, velocity simulations for each axis are conducted for a segment of interpolation points between adjacent machining positions in Table 1. The relevant parameters for this interpolation path segment are provided in Table 2.

**Figure 9:**
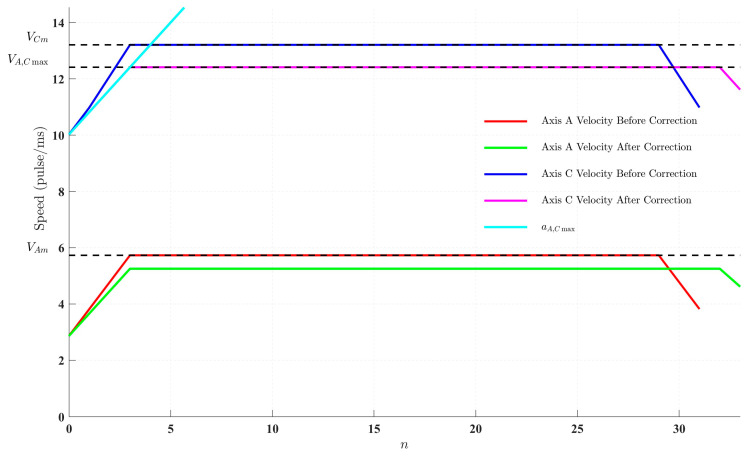
A–C-axis motor rotation speed curve.

The pulse equivalent for the translational axes was set at 1/10,000 mm/pulse, while for the rotational axes, it was set at π/500,000 rad/pulse. The maximum angular velocity *V_A_*_,*Cmax*_ and angular acceleration *a_A_*_,*Cmax*_ of the rotational axes were used as kinematic constraints for velocity planning. The velocity curves of the A-axis and C-axis, both before and after correction, were sampled and are presented in Figure 9. In this figure, *V_Cm_* and *V_Am_* represent the actual angular velocities of the C-axis and A-axis, respectively, with the slope in the acceleration phase indicating the actual angular acceleration.

It can be observed that the angular velocity and angular acceleration of the C-axis initially exceeded the kinematic limits of the machine. After corrections were applied based on the maximum allowable angular velocity and angular acceleration (*V_A_*_,*Cmax*_ and *a_A_*_,*Cmax*_), the C-axis’s angular velocity and angular acceleration were brought into compliance with the kinematic constraints. However, this adjustment also led to a reduction in the feed velocity and maximum permissible acceleration of the translational axes, which, in turn, decreased both the angular velocity and angular acceleration of the A-axis.

**Figure 10:**
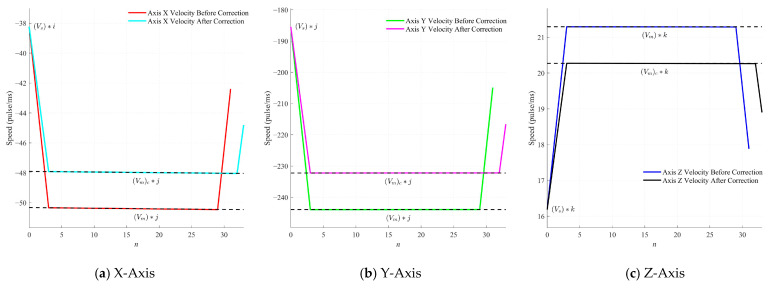
Translational axis motor rotation speed curve.

The corrected velocity profiles for the translational axes are presented in Figure 10. Using Equations (7) and (11), the maximum composite translational velocities (*V_m_*)*_C_* and accelerations (*a_max_*)*_C_*, constrained by the C-axis, were computed to adjust the overall translational velocity *V_m_* and acceleration *a_max_*. These velocities were then allocated to the X, Y, and Z axes based on the unit vector (*i*, *j*, *k*). In the figure, the slope of the acceleration segments illustrates the changes in acceleration before and after the correction for each axis. A positive motor velocity corresponds to counterclockwise rotation, while a negative velocity indicates reverse rotation.

As depicted in Figure 10, the corrected feed velocities and accelerations for each axis were reduced. Nevertheless, this correction effectively mitigates any potential negative impacts on the machine caused by exceeding the C-axis limits.

### 4.3. Real-Time Performance Verification

#### 4.3.1. Response Error

Response error is a critical metric for assessing the real-time performance and accuracy of multi-axis motion control systems. It refers to the discrepancy between the software-calculated time (theoretical time) and the hardware execution time (actual time).

**Figure 11:**
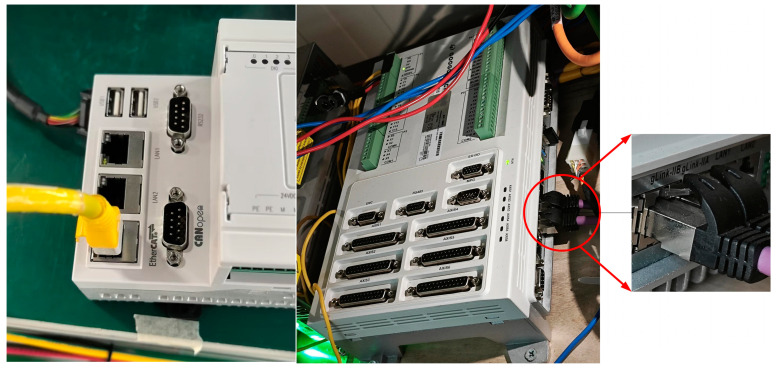
Comparison of motion controllers.

To highlight the advantages of the CNC system developed based on the gLink-II protocol, a comparative analysis of the response errors is conducted against a CNC system developed using the EtherCAT protocol. A comparison between the gLink-II and EtherCAT controllers of the same brand is shown in Figure 11.

**Figure 12:**
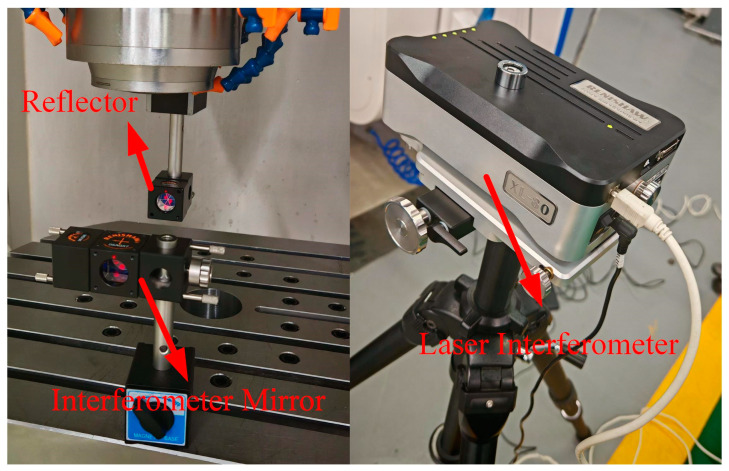
Laser interferometer.

Theoretical time is recorded using the high-precision timer interface, GTN_GetClockHighPrecision, integrated within the motion controller. Actual time is measured in real-time using a laser interferometer, as shown in Figure 12. This interferometer is connected to the FPGA of the motion controller and synchronized with an external clock.

**Figure 13:**
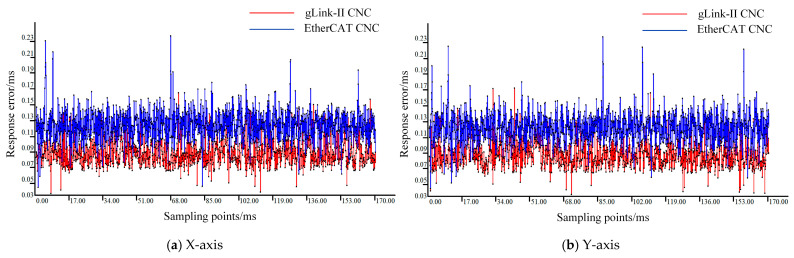

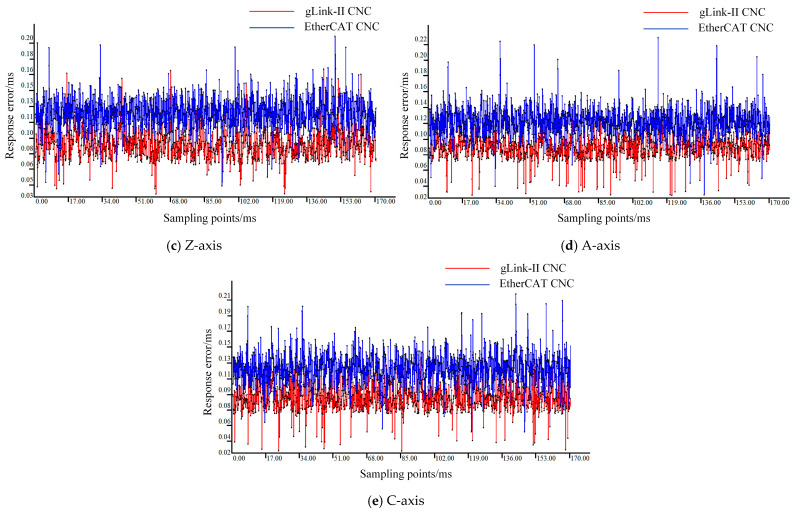
Comparison of response errors between gLink-II and EtherCAT CNC systems.

To ensure machine tool stability, each of the five axes was tested individually with the motion controller. A sampling interval of 0.1 ms was applied to sample ten interpolation path segments. The built-in GTN_GetClockHighPrecision interface was used to record timestamp t_1_ when the motion command was issued. The laser interferometer was set with a threshold of 1 μm, with its inherent delay compensated. The DI channel of the motion controller was configured in event capture mode, such that when the axis motion exceeded the predefined threshold, timestamp t_2_ was recorded upon triggering by the DI channel. The difference between t_1_ and t_2_ is defined as the response error. The response errors for each axis in both CNC systems were measured and are shown in Figure 13.

The maximum, minimum, and average response error data for both the gLink-II and EtherCAT CNC systems are presented in Table 3 and Table 4, respectively.

As illustrated in Figure 13, a comparison of the response errors across each axis between the designed gLink-II CNC system and the EtherCAT CNC system indicates that the response error of the gLink-II system remains around 0.09 ms over a prolonged period, demonstrating both lower overall error levels and faster response to interpolation commands. This highlights the superior performance of the gLink-II CNC system. According to Table 3 and Table 4, the maximum response error of each axis in the gLink-II system does not exceed 0.2 ms, and its average response error is 36–51% lower than that of the EtherCAT CNC system.

#### 4.3.2. Real-Time Performance Within the Interpolation Cycle

The interpolation cycle encompasses several key components: the time required for the interpolation algorithm to process the path planning command and calculate a single interpolation point, the time taken for data transmission to the motion controller, the execution time for motor movement, and the time for receiving feedback signals from the motor. The gLink-II CNC system, developed using the gLink-II communication protocol, significantly reduces data transmission time, as evidenced by the results from adjacent interpolation path segments presented in Table 1.

**Figure 14:**
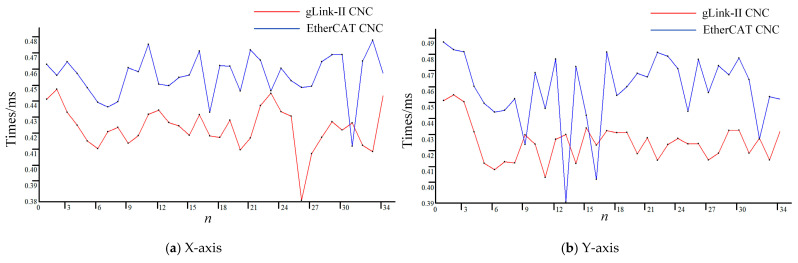

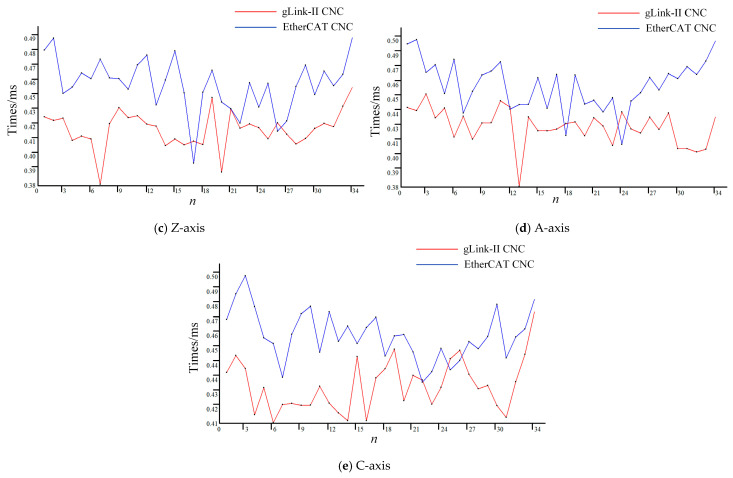
Comparison of interpolation cycle completion time between gLink-II and EtherCAT CNC systems.

To optimize the interpolation process, the thread priority is set to THREAD_PRIORITY_TIME_CRITICAL by calling SetThreadPriority, ensuring the highest level of processing. The GTN_GetClockHighPrecision interface is then used to wait for a 0.5 ms interval. If a timeout occurs, the system records the corresponding timestamp and continues to wait for the interpolation to complete, guaranteeing that the interpolation task operates within a strict 0.5 ms cycle. The internal clock of the controller logs the timestamp, *t*_3_, marking the start of the real-time interpolation algorithm. The displacement within one interpolation cycle for each axis is established as a threshold for the laser interferometer. When this threshold is surpassed, the trigger signal timestamp, *t*_4_, is returned from the DI channel. The time difference between *t_3_* and *t_4_* for each interpolation cycle is illustrated in Figure 14.

As depicted in Figure 14, the interpolation tasks for each axis in the gLink-II CNC system are completed within a time range of 0.38 ms to 0.46 ms for the given path segment. In contrast, using the velocity planning parameters from Table 2, the EtherCAT CNC system experiences interpolation cycle overruns in certain segments. Overall, the interpolation tasks for all axes in the gLink-II CNC system consistently complete within 0.5 ms, fully meeting the real-time requirements of CNC systems.

## 5. Discussion

This study focuses on the system design and experimental validation of a five-axis CNC system, utilizing the gLink-II bus protocol. The system demonstrates significant improvements in both real-time control and motion accuracy. By shifting the interpolation control process to the tool path file stage, the structural openness of the .CLS file is preserved, offering a more flexible solution for complex surface machining.

The system architecture adopts a dual-layer design, integrating Windows with the RTOS, supported by a ring buffer-based data exchange mechanism. This structure ensures effective decoupling between human–machine interaction and core real-time control. Compared to traditional CNC systems, the proposed approach significantly reduces data transmission latency and enhances task scheduling flexibility. The experimental results indicate that the response error across multiple axes is consistently maintained within 0.2 ms, showcasing excellent real-time performance and high control accuracy.

At the algorithmic level, the study introduces a direct six-coordinate interpolation method based on tool position points, which differs from traditional post-processing-based methods. This approach effectively eliminates trajectory distortion typically introduced during post-processing conversions. Additionally, acceleration and deceleration adjustments are made according to the limit speeds and accelerations of the A and C rotary axes. This not only enhances the smoothness of the interpolation path but also ensures operational safety. The experimental comparisons confirm that the gLink-II CNC system completes interpolation calculations and motor execution within a 0.5 ms cycle, fully satisfying real-time control requirements.

Moreover, the data flow mechanism, based on the producer–consumer model, effectively boosts the efficiency of parallel task execution. Particularly in scenarios involving large data volumes and high-frequency task switching, the ring buffer design mitigates issues like blocking due to lock contention, ensuring stable system operation.

Despite these advancements, certain limitations remain. The current system is primarily designed for five-axis machine tools with an A/C dual rotary head structure, and its compatibility with other structural configurations and practical machining experiments requires further exploration. The interpolation algorithm does not yet incorporate complex surface fitting or spline-based strategies, limiting its adaptability to extreme path geometries. Additionally, the system lacks state awareness and feedback control mechanisms, leading to insufficient adaptability under external disturbances. As digital twin technologies continue to evolve, future research could integrate these technologies with AI-driven intelligent algorithms to further enhance CNC system intelligence.

## 6. Conclusions

This paper presents and implements a five-axis CNC system based on the gLink-II bus protocol, designed to offer both high real-time performance and reliability. A direct six-coordinate interpolation model, derived from tool position points, is introduced, alongside an acceleration and deceleration planning algorithm that respects the velocity and acceleration limits of the rotary axes. These innovations optimize trajectory calculation and motion control during the machining of complex parts. The system adopts a layered architecture: the Windows operating system manages user interaction, while the RTOS handles core tasks in real time, ensuring seamless data exchange and rapid system responses.

The experimental results validate the exceptional real-time capabilities and precise motion control of the system in practical settings. The response error for all axes in the gLink-II CNC system remains consistently under 0.2 ms, with an average error of less than 0.09 ms. This represents a 36–51% improvement over the EtherCAT CNC system. The interpolation cycle stays below 0.5 ms, and the integration of velocity planning further enhances machine operation smoothness. These characteristics satisfy the stringent demands for high-efficiency and high-precision control in CNC systems, providing a novel approach to advancing the development of five-axis CNC systems.

## Figures and Tables

**Table 1:** Data of adjacent machining points.

Segment Number	*x*/mm	*y*/mm	*z*/mm	*i*	*j*	*k*
24	35.164	−440.607	−91.961	−0.201263	−0.975827	0.085182
25	34.946	−440.586	−91.755	−0.201849	−0.975709	0.085144

**Table 2:** Other parameters.

Parameters	*T*/mm	*V_max_*/(mm/s)	*V_s_*/(mm/s)	*V_e_*/(mm/s)	*a_max_*/(mm/s²)	*V_A, Cmax_*/(rad/s)	*a_A, Cmax_*/(rad/s²)
Value	0.5	25	19	21	5500	0.078	22

**Table 3:** Response error data of each axis in the gLink-II CNC system.

Axis	Maximum Error (ms)	Minimum Error (ms)	Average Error (ms)
X	0.15586	0.03631	0.08407
Y	0.16448	0.03445	0.08139
Z	0.16173	0.03009	0.07925
A	0.12959	0.02124	0.07231
C	0.12960	0.02073	0.07032

**Table 4:** Response error data of each axis in the EtherCAT CNC system.

Axis	Maximum Error (ms)	Minimum Error (ms)	Average Error (ms)
X	0.22511	0.04044	0.12587
Y	0.22704	0.03842	0.11080
Z	0.19660	0.04360	0.11423
A	0.21414	0.02081	0.10926
C	0.20653	0.04287	0.09751

## Data Availability

The data presented in this study are available on request from the corresponding author.
